# The Global Fund's Bangladesh program

**DOI:** 10.1016/j.ijregi.2025.100618

**Published:** 2025-03-04

**Authors:** Md Ferdous Rahman, Md Sharful Islam Khan, M Mamun Huda, Utpal K. Mondal, Muhammad J.A. Shiddiky, Allen G. Ross

**Affiliations:** 1 Rural Health Research Institute, Charles Sturt University, Orange, Australia; 2 Programme for HIV and AIDS, Health Systems and Population Studies Division, International Centre for Diarrhoeal Diseases Research, Dhaka, Bangladesh

**Keywords:** Global fund, HIV, Tuberculosis, Malaria, Bangladesh

## Abstract

The Global Fund program, launched in 2002, focuses on combating HIV/AIDS, tuberculosis (TB), and malaria. To date, it has mobilized over US$ 65 billion across 120 countries, contributing to saving more than 65 million lives. In Bangladesh, the Global Fund has played a pivotal role in expanding treatment coverage, enhancing case detection, and strengthening prevention efforts. Despite notable progress, significant challenges persist, particularly among high-risk populations such as people who inject drugs, sex workers, and transgender individuals. These groups face stigma and limited access to healthcare, both of which hinder disease control. Additionally, drug resistance presents a growing threat to the control of malaria and TB. Looking ahead, continued support from the Global Fund will be critical, particularly in scaling up diagnostic infrastructure, promoting community-led interventions, and enhancing cross-border collaboration. These efforts are essential to ensuring sustained progress and achieving the long-term goal of eliminating HIV, TB, and malaria in Bangladesh. Effective coordination among government agencies, non-governmental organizations, and international donors will be essential to ensure the success of these initiatives.

## Highlights

• The Global Fund has been working in Bangladesh since 2003.
• The country has improved HIV targets with 75% on antiretroviral therapy, and 90% suppressed.
• Between 2010 and 2020 tuberculosis cases surged by 50% while mortality was halved.
• Between 2018 and 2021 the malaria program distributed 3.7 million insecticidal bed nets.
• Key challenges: Injectable drug users/men who have sex with men, drug-resistant tuberculosis/malaria, and poor healthcare access.

## The Global Fund

Since its establishment in 2002, the Global Fund to Fight AIDS, Tuberculosis, and Malaria (GFATM) has transformed global health efforts against three of the most formidable infectious disease threats [1]. Through mobilizing resources and fostering strategic partnerships, the Global Fund has saved over 65 million lives and significantly improved health outcomes in the hardest-hit regions, reshaping the global fight against these critical diseases [2]. With over US$ 65 billion invested across more than 120 countries, the Fund has not only saved millions of lives but also strengthened social and economic infrastructures. Each life saved generates a ripple effect, empowering families, communities, and nations in their ongoing fight against these devastating diseases [1,2].

Aligned with its mission to eliminate HIV/AIDS, tuberculosis (TB), and malaria while strengthening health systems, the Global Fund also supports the sustainable development goals (SDGs), particularly SDG 3, which aims to promote global health and well-being. As of 2024, the Global Fund provides 29% of international funding for HIV programs, 76% for TB, and 62% for malaria. In the countries it supports, disability-adjusted life years for these diseases have dropped by 56% from 2000 to 2021, reflecting fewer deaths, reduced disabilities, and significant quality-of-life improvement for millions [2].

The Global Fund's funding model prioritizes country ownership and inclusive decision-making through country coordinating mechanisms, which bring together not only governments but also communities, vulnerable populations, civil society, and the private sector. This collaborative approach empowers countries to lead their response to HIV, TB, and malaria by allocating resources based on local needs, ensuring that interventions are impactful, tailored, and sustainable. This model effectively addresses areas of greatest impact, supporting long-term progress in global health [3].

## Global Fund commitment to Bangladesh

The Global Fund has partnered with Bangladesh since 2003 [4]. With a population exceeding 172 million, Bangladesh faces a substantial burden of TB, a concentrated HIV epidemic among key populations (KPs) (e.g., people who inject drugs [PWID], men who have sex with men [MSM], male sex workers, female sex workers [FSW], and transgender women, who are locally known as hijra [TGW]) and ongoing malaria transmission along the Myanmar border [[5], [6], [7]]. As of 2022, the Global Fund had signed grants totaling over US$ 835 million with Bangladesh and disbursed over US$ 700 million [8]. In December 2023, the Economic Relations Division of Bangladesh signed three new grant agreements with the Global Fund, valued at US$ 80.68 million, to bolster efforts against HIV/AIDS, TB, and malaria, while further strengthening Bangladesh's healthcare infrastructure [9].

The impact of the Global Fund's investment in Bangladesh is evident. According to the 2021 Global Fund audit, 98% of allocated funds in Bangladesh were effectively utilized, yielding substantial advancements in TB case detection, malaria prevention, and an increase in the number of people living with HIV on treatment. However, the report highlighted the need for continued financial support, especially in remote and underserved regions, to sustain progress and ensure health outcomes across the country [8].

This review synthesizes information on the Global Fund's contributions to combating HIV, TB, and malaria in Bangladesh. A comprehensive electronic search was conducted using keywords such as ‘Global Fund’, ‘HIV’, ‘tuberculosis’, ‘malaria’, ‘harm reduction’, and ‘Bangladesh’ to identify relevant sources. We reviewed peer-reviewed articles, national program reports, and strategic plans from the AIDS and STD Programme (ASP), the National Tuberculosis Control Programme (NTP), and the National Malaria Elimination Programme (NMEP). Additionally, reports from the Global Fund, World Health Organization (WHO), BRAC, and other international development partners were analyzed to assess program achievements and challenges. This review follows a descriptive approach, summarizing key achievements, challenges, and future directions of Global Fund-supported programs in Bangladesh.

## The triple threat to Bangladesh's healthcare system

Despite considerable progress, HIV, TB, and malaria continue to pose significant challenges to Bangladesh's healthcare system, straining resources and impacting economic stability. Although global incidence rates have declined, these diseases persist in Bangladesh, disproportionately affecting high-risk and marginalized populations. TB remains one of the leading causes of death by an infectious disease. While HIV prevalence remains low, it is concentrated among KPs (1-4%), exacerbated by limited healthcare access, stigma, and discrimination. Malaria, while controlled in many parts of the country, persists in the southeastern border, where vector control challenges, drug resistance, and climatic factors sustain transmission hotspots. These three diseases not only burden the healthcare system by overwhelming diagnostic, treatment, and prevention capacities but also exacerbate poverty, highlighting the critical need for sustained and integrated public health interventions to reduce their impact [6,7,10].

## HIV in Bangladesh

Since the first reported case in 1989, Bangladesh has maintained a low HIV prevalence (<0.1%) among the general population. However, the epidemic is increasingly concentrated within KPs [7,11]. The most recent integrated biological and behavioral survey (IBBS), conducted in 2020, reported an overall HIV prevalence of 2.3% among KPs, with the highest rates observed among PWID at 4.1%, followed by MSM at 1.5 %, TGW at 0.9%, and FSW at 0.1%. Among these groups, HIV prevalence among MSM aged 25 years and above was 2.4% compared to 0.9% among those under 25, highlighting a significant burden among older adults within this subpopulation [12].

**Figure 1:**
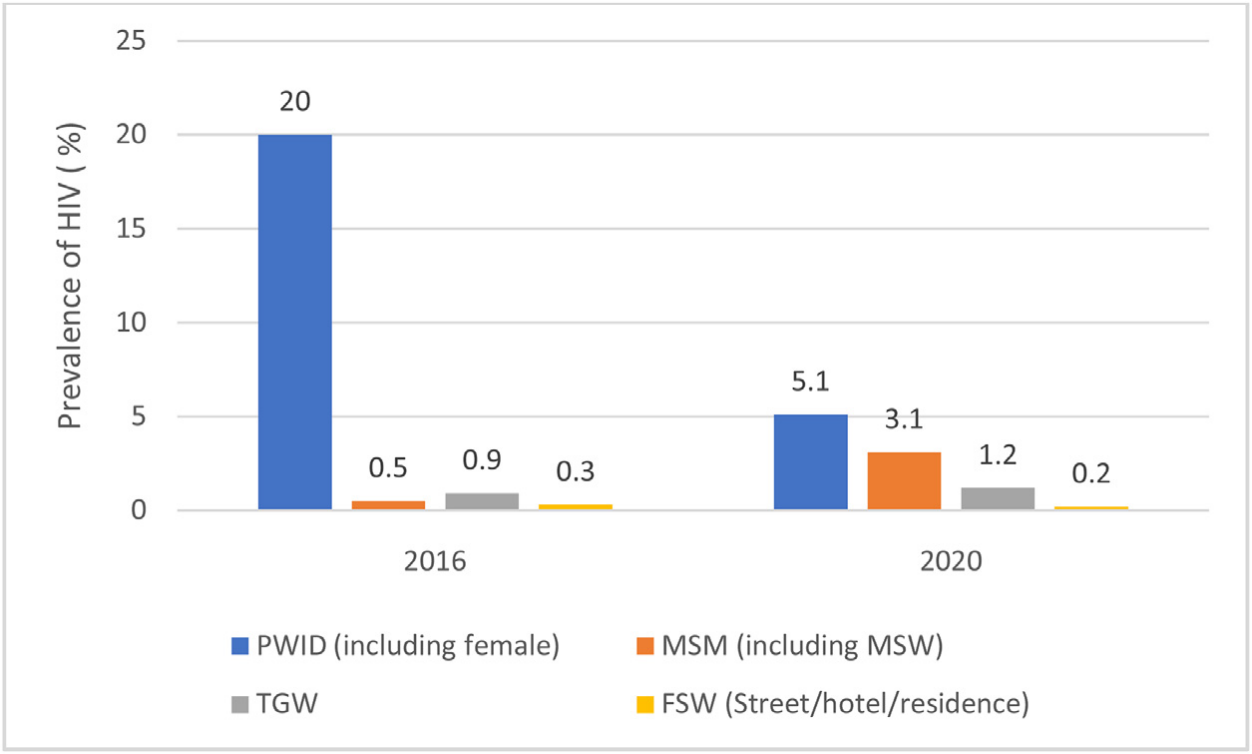
Prevalence of HIV in Dhaka in 2016 and 2020. Data source: The Global Fund Bangladesh - HIV funding request 2023. FSW, female sex workers; MSM, men who have sex with men; PWID, people who inject drugs; TGW, transgender women, who are locally known as hijra.

Geographical disparities are also pronounced, with urban centers, particularly Dhaka (the nation's capital), showing notably higher prevalence rates. In Dhaka, PWID recorded a prevalence of 5.1%, MSM at 3.1%, TGW at 1.2%, and FSW at 0.2%, reflecting urban hotspots of core transmission (Figure 1) [13]. As shown in Figure 1, the IBBS (2020) reported that MSM prevalence increased from 0.5% in 2016 to 3.1% in 2020, and TGW from 0.9% to 1.2%, while FSW prevalence remained low and stable. There was also a sharp decline in PWID prevalence in Dhaka, from 20% in 2016 to 5.1% in 2020 [12,13]. However, recent data suggests an upward trend, indicating changes in HIV transmission patterns over time. In 2023, 1276 new HIV cases were reported, bringing the cumulative total to 10,984 since 1989. Among these newly reported cases, the majority were from the general population (27.7%), followed by MSM (18.2%), migrants (17.6%), and PWID (13.1%) [14]. Furthermore, the AIDS epidemic model predicts that MSM may play an increasingly significant role in the HIV epidemic in Dhaka and throughout the country [15].

## Addressing the HIV burden in Bangladesh

Even before detecting its first HIV case, the Government of Bangladesh (GoB) took a proactive approach by establishing the National AIDS Committee in 1985, anticipating a potential epidemic. This early initiative laid the foundation for a structured national response, leading to the formation of the National AIDS/STD Programme (NASP) in 1997, which oversees prevention, treatment, care, and support efforts. That same year, Bangladesh became the first country in the region to adopt a comprehensive national policy on HIV/AIDS and sexually transmitted infections, followed by successive National Strategic Plans to guide interventions [16].

HIV prevention programs for KPs were initiated in the mid-1990s, with financial support from the World Bank, UNICEF, UNAIDS, and the Global Fund, significantly expanding HIV services. These interventions have primarily been implemented through non-governmental organization partnerships under NASP's leadership, demonstrating Bangladesh's commitment to a multi-sectoral approach. Currently, the Global Fund is the primary financial supporter of Bangladesh's HIV response, covering 80% of prevention funding and fully supporting program management costs. The three principal recipients of Global Fund grants—GoB, Save the Children, and icddr,b—oversee program implementation directly and through sub-recipients, ensuring continuity [17].

As a result of these sustained efforts, Bangladesh has made substantial progress in expanding HIV care services. As of 2023, progress toward the 95-95-95 targets shows that 73% of people living with HIV know their status, 75% of diagnosed individuals are on antiretroviral therapy (ART), and 90% of those on ART have achieved viral suppression. These improvements reflect the success of community-based approaches, peer-led outreach, and the integration of KP services within the public healthcare system [14].

With the support of the Global Fund, the International Centre for Diarrhoeal Disease Research, Bangladesh (icddr,b) has introduced several ICT-based interventions to expand HIV prevention services for marginalized populations. These initiatives use mobile applications, SMS, and web-based platforms to enhance healthcare access for gender-diverse and sexually diverse populations who may hesitate to seek care in traditional healthcare settings. In 2023, the icddr,b also launched a social media-based intervention for HIV and sexually transmitted infection prevention, initially targeting 5000 participants. The program's success led to its expansion, ultimately reaching 10,000 individuals within marginalized communities, thereby significantly improving healthcare access for hidden high-risk groups.

In addition to these digital advancements, icddr,b piloted a pre-exposure prophylaxis (PrEP) initiative for MSM and male sex workers in Dhaka. By December 2023, 232 clients had initiated PrEP. Baseline assessments indicated high-risk behaviors among participants, but follow-up data showed high retention rates, improved condom use, better mental health, and notably, no new HIV infections among those who consistently used PrEP during the 22-month intervention [18].

The Global Fund's contributions have significantly boosted the number of people living with HIV on treatment, with ART coverage now reaching 89.11% in 2023. Retention rates among KPs have also improved markedly; for example, retention among PWID increased from 16% in 2015 to 87% in 2020, with similar gains among MSM and TGW individuals. Efforts to expand prevention coverage have also yielded positive results, with intervention coverage among PWID increasing from 29% in 2018 to 52% in 2021 [8].

## Tuberculosis in Bangladesh

**Figure 2:**
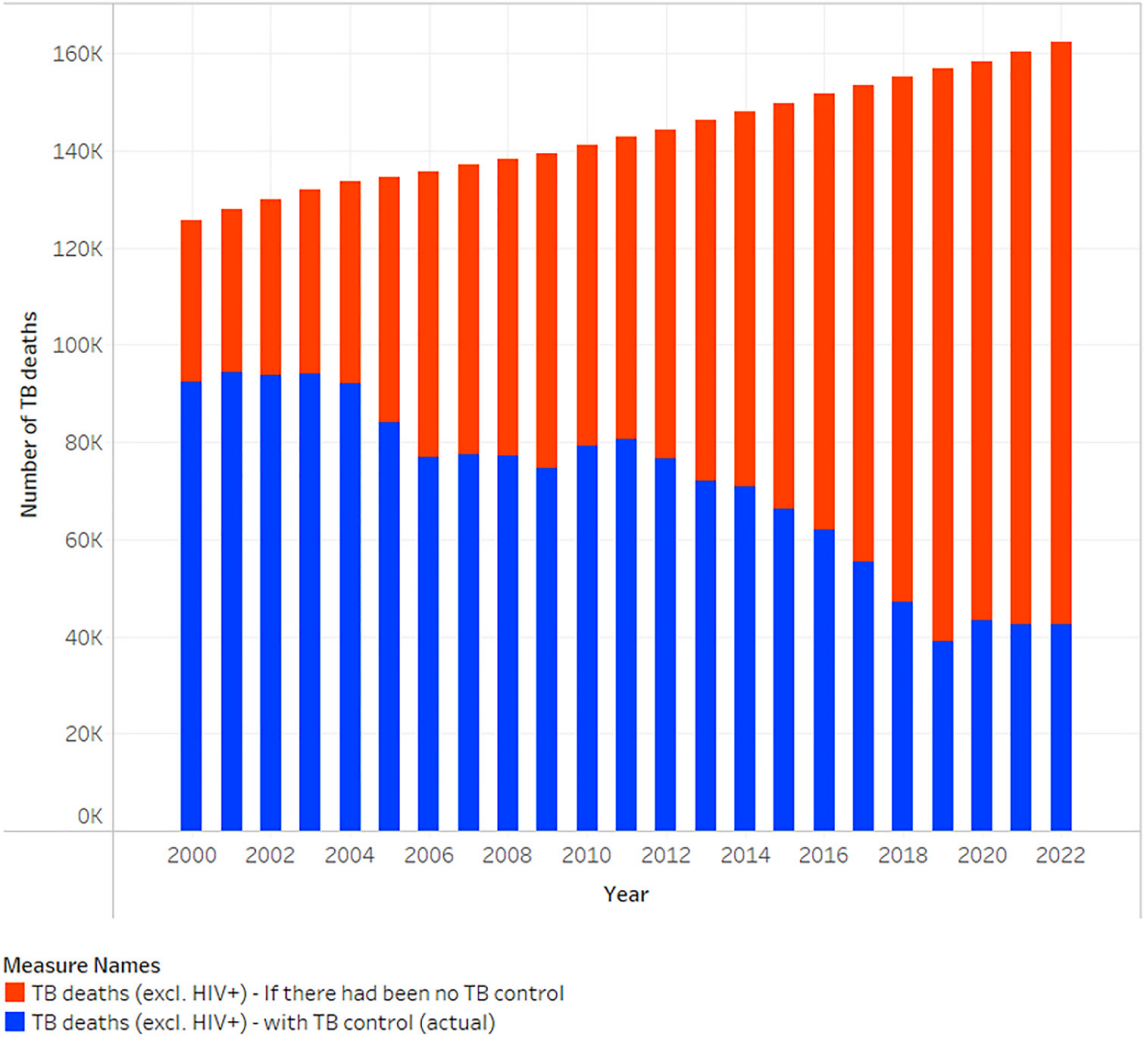
Impact of TB control measures on TB mortality (2000–2022). A comparison of actual TB deaths versus projected deaths without control in Bangladesh. Data source: The Global Fund Report, 2024. TB, tuberculosis.

Bangladesh is ranked the 7th highest TB burden country globally, accounting for 3.6% of the world's TB cases, with an incidence rate of 221 per 100,000. In 2022, TB caused approximately 42,000 deaths annually (Figure 2), with a mortality rate of 24 per 100,000, making TB the deadliest infectious disease in the nation [19,20]. That same year, the country reported an estimated 379,000 new TB cases, achieving a 69% case notification rate with 261,957 notified cases, of which 75% were bacteriologically confirmed, highlighting ongoing challenges in TB control [20,21]. Furthermore, the WHO has designated Bangladesh as a high-burden country for both multidrug-resistant/rifampicin-resistant TB (MDR/RR-TB) for the 2021-2025 period, reflecting the critical need for enhanced TB management and intervention strategies [22].

## Addressing the tuberculosis burden in Bangladesh

Bangladesh has achieved significant advancements in TB control, markedly improving case detection and treatment outcomes through strategic interventions and substantial support from the Global Fund and Stop TB Partnership. Between 2010 and 2020, TB case notifications surged by 50%, while mortality rates were halved, highlighting major improvements in disease management. Community-based interventions have been particularly effective, with 54% of TB cases detected through these efforts in 2021, up from 49% in 2018, reflecting the success of expanded outreach initiatives. The country achieved an impressive 85% treatment coverage rate for drug-sensitive TB, maintaining a treatment success rate of 97% in 2020 [8].

The NTP has developed a hierarchical network of TB laboratories, ranging from peripheral facilities to regional and national TB reference laboratories, which has significantly expanded diagnostic capacity. By 2024, Bangladesh had expanded its capacity to 735 GeneXpert machines across 627 sites and 150 TrueNat machines, up from 622 GeneXpert and 38 TrueNat machines operational in December 2023 [23].

In 2022, RR-TB testing was conducted on 37% of new pulmonary TB cases and all previously treated cases, with 99% of the 1,283 patients diagnosed with RR and MDR-TB promptly initiating treatment [20]. The country is also rolling out an electronic case-based recording and reporting system, e-TB Manager, which has significantly improved patient management and improved data accuracy. The Global Fund's support has been crucial in scaling up diagnostic tools and adapting services to better reach vulnerable populations, including those displaced by climate-related factors in urban [8].

## Malaria in Bangladesh

Malaria has long been a significant public health problem in Bangladesh, particularly in the southeastern border corridor. Most cases are concentrated in three high-endemic districts (e.g., Bandarban, Rangamati, and Khagrachari) collectively known as the Chattogram Hill Tracts. Over the past 2 decades, the country has made impressive progress toward malaria elimination (Supplementary Figure 1), achieving a 93% reduction in malaria cases, and a 94% decrease in malaria-related deaths between 2008 and 2020 [24]. As of now, 51 out of Bangladesh's 64 districts are free from malaria, with pre-elimination efforts underway in the remaining 10 districts [25].

In Southeast Asia's Greater Mekong Subregion, the spread of multidrug-resistant malaria along the Myanmar-Thailand border poses a significant challenge. Myanmar, a significant contributor to malaria resistance in the region, experienced a surge in cases from 78,000 to 584,000 between 2019 and 2022 [26]. The widespread resistance of *Plasmodium falciparum* to artemisinin-based combination therapies complicates the situation, necessitating intensified surveillance and treatment adaptations [27]. This rise has increased local transmission in Thailand and is further exacerbated by displaced populations, including refugees seeking medical care across the border. The cross-border transmission of resistant strains poses a direct threat to Bangladesh, especially with the migration of forcibly displaced Myanmar nationals, increasing the risk of reintroducing and spreading multidrug-resistant strains along the borders [26,28,29].

## Addressing the burden of malaria in Bangladesh

The NMEP, supported by international partners such as the Global Fund, WHO, and BRAC has implemented several impactful interventions. Between 2018 and 2021, the program distributed 3.7 million long-lasting insecticidal nets to protect at-risk populations, including forest workers, agricultural communities, and children under 5 years. The expansion of rapid diagnostic tests and artemisinin-based combination therapies has been key in reducing malaria transmission. These efforts have strengthened malaria control by improving outreach, education, and case management, especially in remote and underserved areas [22,23]. Surveillance systems have been enhanced, with efforts to implement innovative technologies like ultra-portable diagnostic tools to detect and manage cases more effectively [7].

## Key challenges remaining

Despite significant progress, Bangladesh's efforts to combat HIV, TB, and malaria face several shared structural and systemic challenges. Notably, limited healthcare infrastructure and inadequate access to diagnostic and treatment services persist, especially in remote and hard-to-reach areas [30]. Furthermore, coordination between governmental and private sectors is often fragmented, and there is a lack of integration of community-driven approaches [8]. These shortcomings collectively impair the efficiency and reach of disease control efforts. Additionally, stigmatization and socio-cultural barriers, particularly among marginalized populations, further exacerbate access issues, highlighting the need for sustained and comprehensive interventions to improve the overall capacity of the health system [31].

Shifting focus to HIV, the Office of the Inspector General's audit of Global Fund grants in Bangladesh, published in 2022, identifies major challenges in HIV control, primarily due to insufficient funding, limited-service coverage, and operational inefficiencies. A funding gap of US$ 167 million restricts the expansion of HIV services, with Global Fund-supported efforts active in only 23 high-prevalence districts. Government funding delays further limit services in the remaining 41 districts. Moreover, only 28 HIV counseling and testing centers exist in high-prevalence areas, with just four in Dhaka (i.e., with a population >20 million in metro Dhaka), severely hindering early diagnosis and care for KPs [8].

Operational and logistical barriers further weaken the HIV response. KP interventions, including those for FSW and PWID, often face delays due to slow bureaucratic processes and logistical challenges, such as delayed distribution of HIV test kits and stock-outs of critical testing cartridges. Only 33% of people on ART receive routine viral load monitoring, significantly below global standards, due to the country's limited capacity with only 11 viral load testing sites [8,13]. Furthermore, stigma, discrimination, and socio-legal barriers significantly exacerbate the challenges in addressing HIV among KPs. High levels of stigma and punitive laws against same-sex relations, drug use, and sex work deter marginalized groups from accessing HIV prevention and treatment services. Consequently, the coverage of preventive interventions remains alarmingly low among these groups. In 2021, only 52% of PWID and 25% of MSM were reached by services, falling far short of UNAIDS’ recommended 80% coverage target [8].

Similarly, Bangladesh's fight against TB is plagued by significant challenges in case detection and treatment accessibility, particularly in rural areas. Although diagnostic technologies like GeneXpert have been expanded, underutilization and equipment malfunctions remain recurring issues. These operational challenges are compounded by chronic shortages of essential health supplies, weakening overall program performance [8,32]. With a case notification rate of 69%, approximately three out of every 10 TB cases are missed, leaving a substantial number of people untreated and enabling ongoing transmission within communities. This shortfall is particularly evident in drug-resistant TB notifications, where only 1283 cases were reported against the United Nations High-Level Meeting target of 5400, highlighting the urgent need for improved case detection and reporting mechanisms [20,33].

Delays in diagnosis and treatment initiation further hinder TB control efforts. A study published in 2019 revealed that the average delay from symptom onset to diagnosis is 77 days, with 57 days of that delay attributable to patients’ reluctance to seek care promptly [34]. The coexistence of formal and informal healthcare providers in Bangladesh's pluralistic health system exacerbates this issue, as many patients initially turn to informal providers, such as village doctors or drug sellers. The widespread illegal dispensing of antibiotics by drug sellers, combined with their limited knowledge, leads to irrational antibiotic use, further contributing to antimicrobial resistance and delayed TB diagnosis and care. This poorly structured referral system between informal and formal healthcare sectors contributes to significant delays in diagnosis and care [32]. Additionally, domestic funding for TB dropped from 31% in 2021 to 11% in 2022, further straining the healthcare system. These barriers, coupled with a poorly structured referral system and workforce shortages, continue to hamper Bangladesh's efforts to effectively control TB [8,33].

Malaria elimination in Bangladesh is impeded by a confluence of geographical, socio-cultural, and systemic challenges that undermine current efforts. As mentioned, most incident cases are concentrated in the Chattogram Hill Tracts, which account for 95% of the country's malaria burden. These areas face distinct difficulties, including rugged terrain, limited healthcare access, and cross-border transmission from neighboring countries like Myanmar and India. The forested landscapes complicate access to essential preventive measures, such as distribution of insecticide-treated nets and timely diagnosis and treatment services. Cross-border movement further exacerbates the situation by importing cases that fuel ongoing transmission cycles. Additionally, the emergence of insecticide resistance among mosquitoes and antimalarial drug resistance threatens the effectiveness of current interventions [6,25].

## Future strategies

To sustain and enhance progress against HIV, TB, and malaria in Bangladesh, continued support from the Global Fund remains essential. However, achieving long-term sustainability also requires diversifying funding sources, including increased domestic investment and partnerships with other international organizations. Domestic resource mobilization and reducing dependence on external funding will empower Bangladesh to maintain progress independently. A critical focus for the Global Fund should be on expanding diagnostic capacity in underserved regions to promote early detection and treatment through tools like GeneXpert for TB and rapid diagnostic tests for malaria. Strengthening community-based interventions for KPs and vulnerable rural groups will make prevention and care efforts more inclusive. Additionally, cross-border collaboration, particularly for malaria control, is crucial to mitigate transnational disease transmission and ensure cohesive control efforts across borders.

An effective strategy must address barriers across institutional, community, and individual levels. Institutionally, healthcare systems need robust supply chains, improved drug access, streamlined treatment protocols, and patient-centered services to overcome systemic challenges. At the community level, addressing stigma, discrimination, and misinformation through targeted awareness campaigns will help foster supportive environments for affected individuals. Individual-level strategies should focus on addressing financial barriers, supporting treatment adherence, and enhancing social support for those facing limited family resources.

Considering lessons learned from the COVID-19 pandemic, building crisis preparedness and resilience within the healthcare system is critical to tackling future health emergencies. Strengthening multi-sectoral collaboration and community engagement, with a focus on policy advocacy and removing legal barriers, will expand the reach and effectiveness of disease control programs. Additionally, investing in digital health interventions and data science will improve health monitoring, decision-making, and adaptability, making Bangladesh's healthcare system more resilient and responsive to evolving public health challenges in the 21^st^ century.

## Declarations of competing interest

The authors have no competing interests to declare.

## Appendix Supplementary materials

Image, application 1

## Footnotes

Supplementary material associated with this article can be found, in the online version, at doi:10.1016/j.ijregi.2025.100618.
