# Supplementary material for: The Global Fund's Bangladesh program
Source: IJID Reg. 2025 Mar 4;15:100618. doi: 10.1016/j.ijregi.2025.100618 (PMC11964771; doi:10.1016/j.ijregi.2025.100618)
Supplement: Supplementary file 1 [file mmc1.docx]

Data source: The Global Fund Report, 2024.

Supplementary Figure 1. Malaria cases and deaths: 2008 – 2022.

Data source: The Global fund Bangladesh- Malaria funding request 2023.
